# Effects of Two Chlorine Gas Attacks on Hospital Admission and Clinical Outcomes in Kafr Zita, Syria

**DOI:** 10.7759/cureus.17522

**Published:** 2021-08-28

**Authors:** Abdallah M Elsafti Elsaeidy, Osama I Alsaleh, Gerlant van Berlaer, Abdulrahman A Alhallak, Saad S Saeed, Ashraf Soliman, Ives Hubloue

**Affiliations:** 1 Research Group on Emergency and Disaster Medicine, Vrije Universiteit Brussel, Brussels, BEL; 2 Emergency Department, Hamad Medical Corporation, Doha, QAT; 3 Center for Research and Training in Disaster Medicine, Humanitarian Aid, and Global Health (CRIMEDIM), University of Eastern Piedmont (Università del Piemonte Orientale), Novara, ITA; 4 Disaster Medicine, Al-Sham Humanitarian Foundation, Istanbul, TUR; 5 Research Group on Emergency and Disaster Medicine, Vrije Universiteit Brussel, Brussel, BEL; 6 Faculty of Nursing, Al-Baath University, Hama, SYR; 7 Pediatrics, Hamad Medical Corporation, Doha, QAT; 8 Paediatrics, Hamad Medical Corporation, Doha, QAT; 9 Department Emergency Medicine - Research Group on Emergency and Disaster Medicine, Universitair Ziekenhuis Brussel - Vrije Universiteit Brussel, Brussels, BEL

**Keywords:** syria, health in conflict, chemical attack, chlorine gas, environmental toxicology

## Abstract

Background

In 2014, Hama Governorate was exposed twice to chlorine gas, with 15 patients presenting to Kafr Zita Hospital in Northwest Syria. This study aimed to describe clinical manifestations of chlorine gas exposure to identify factors leading to facility admission and the need for ICU/intubation in conflict-affected areas with limited healthcare infrastructure.

Methods

We conducted a case-series study, using medical records of suspected chlorine-exposed patients presenting at Kafr Zita Hospital on either 11 April or 22 May 2014. Data on age, sex, initial clinical presentation, therapeutic management, and outcome were compared by hospital admission/non-admission and attack date. All patients provided verbal informed consent.

Results

Fifteen patients with signs of chlorine gas exposure had detailed medical records. The mean age was 25.7 years (range 2-59), eight were male (53%), and three (20%) were under age 16.

At initial presentation, all experienced respiratory distress, due to severe airway inflammation confirmed by nonspecific pulmonary infiltrates on chest x-ray, and similar intestinal, neurological, dermatological, ophthalmological, and psychological signs and symptoms. Acute management consisted of oxygen and bronchodilators for all patients, hydrocortisone (93%), antiemetics (80%), and dexamethasone (13%). Seven (47%) made a rapid symptomatic recovery and were discharged the same day and eight (53%) were admitted for a median of two days (range 1-6 days), one of whom required intubation and later died. The only significant associations found were higher mean pulse rate (i.e. 138 versus 124; p=0.043) and body temperature (37.0 versus 36.5; p=0.019) among admitted patients compared to non-admitted.

Conclusion

Our results demonstrated that even in low-resource humanitarian settings the survival rate for chlorine gas exposed patients is fair. Despite the small sample, this study provides insight into the clinical presentation, management, and outcomes of weaponized chlorine gas exposure, though further research is required to understand any chronic consequences.

## Introduction

In March 2011 peaceful popular demonstrations began in Syria which escalated later into an armed protracted conflict [1,2]. One of the weapons used by the Syrian government was chemical weapons multiple times, which has been condemned by the UN Security Council and considered a violation of the international convention on the prohibition of chemical weapons [3]. Chemical weapons were used in Syria as early as December 2012, when civilians in Homs were treated for symptoms of chemical exposure. The use of chemical weapons escalated beginning in March 2013, particularly in Rural Damascus [4]. After the chemical attack on civilians in Homs, 87 Human chemical weapons attacks have been recorded, several involving chlorine gas [5]. The Syrian government agreed to destroy all of its chemical weapons after the deadly chemical attack on Ghouta in 2013, however, a sudden increase of reports on chemical strikes was recorded with a peak in April 2014 [6].

The use of chemical weapons, including chlorine gas, is prohibited internationally by the 1925 Geneva Gas Protocol and constitutes a war crime [7]. The use of chemical weapons in Syria has often been looked at as a problematic issue [8]. A number of governmental and non-governmental international organizations in Syria have reported evidence of chemical weapons attacks in the form of toxic gas such as Sarin, chlorine, and mustard agents. The Organization for the Prohibition of Chemical Weapons (OPCW) along with the United Nations (UN) confirmed the use of chemical weapons. The Syrian American Medical Society (SAMS) carried out a parallel investigation that involved the collection of patients' biological samples and environmental samples after gas attacks. SAMS confirmed the findings of OPCW-UN investigations [3].

Chlorine is a common toxic inhalant and potent eye, skin, and respiratory irritant [9]. At room temperature, it is a moderately water-soluble yellow-green gas, over twice as heavy as air with a detectable odor at low concentrations. Chlorine reacts with water in human mucous membranes and airways to form hydrochloric and hypochlorous acids, leading to acute inflammation of the conjunctiva, nasal mucosa, pharynx, larynx, trachea, and bronchi. Acute exposures may result in nonspecific clinical symptoms of acute airway obstruction including wheezing, cough, rales, chest tightness, dyspnoea, and hypoxemia. Chest radiographs may show infiltrative lesions [10]. Symptoms may vary by degree of exposure (e.g., acute low levels, acute high levels), with higher concentrations potentially contributing to acute respiratory distress syndrome (ARDS), pulmonary edema, pulmonary inflammation with or without infection, respiratory failure, and death [11-13]. Reducing lung injury and improving respiratory function are the principal therapeutic aims for exposed patients [14]. Chlorine has been weaponized since 1915 [15]. with widespread casualties described in Iraq since January 2007 and Syria since 2012 [13,16].

Patients from two chemical attacks in Kafr Zita northern Syria are included [17]. The availability of academic studies around the standard procedures of timely management, civilian protection, and understanding public health consequences of chemical weapons is limited [3]. This paper should reduce the uncertainty around this topic.

This study aimed to examine clinical manifestations of chlorine gas exposure among patients presenting at Kafr Zita Hospital. Objectives were to (i) analyze associations between facility admission and patient demographics, symptoms, treatment, and outcomes; (ii) identify associations between attack date and patient demographics, symptoms, treatment, and outcomes; and (iii) consider treatment approaches for conflict-affected areas with limited healthcare infrastructure.

## Materials and methods

Study design 

We chose a retrospective case-series study design, using medical records of suspected chlorine-exposed patients. 

Setting

Kafr Zita was an opposition-controlled town 30 kilometers north of Hama and has reportedly suffered hundreds of conventional attacks as well as at least seventeen chemical attacks since the Syrian conflict began [18]. Its original population of approximately 18,000 residents has decreased due to conflict. Kafr Zita Hospital (formerly Western Hospital before Eastern Hospital was destroyed in 2014) is a 20-bed facility (Appendix 1), including three intensive care beds and a clinical staff of three doctors and 13 nurses (Appendix 2). Staff were trained by the North Atlantic Treaty Organization (NATO) chemical, biological, radiological, and nuclear (CBRN) Task Force and experienced in disaster management and managing patients exposed to chemicals [19].

In 2014 there were two successive attacks that happened, with the first reportedly causing more injuries and disseminating chemicals more widely than the second [20]. The first occurred on 11 April 2014, between 18:00-19:00, when an improvised barrel bomb was dropped by helicopter, affecting approximately 100 people, five seriously injured, of whom three died. the cases were distributed to different health centers, Kafr Zita was the nearest and it was one of them, it received seven cases of this attack and all were admitted [20,21].

The second occurred on 22 May 2014 at 22:00 hours, injuring approximately 38 people, none of whom died, just like the first attack, the cases were distributed to different health centers, of whom eight cases presented to Kafr Zita Hospital, one of them was admitted [17]. All presented with similar symptoms, indicative of chlorine gas exposure [22,23].

**Figure 1:**
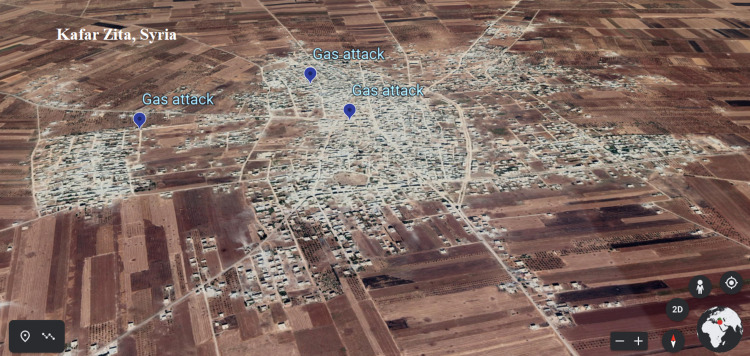
Locations of chlorine-exposed areas in Kafr Zita [21]

Participant eligibility 

Eligibility criteria included patients with chlorine toxidrome, from the area under attack, within the time frame of three hours after the strike. Fifteen patients met these criteria of possible chlorine gas exposure and all provided verbal informed consent. Diagnoses of exposure were based on patient history and physical examination by a CBRN-trained physician (locally trained). Eligibility criteria additionally included a complete medical record with initial presentation, evolution, management, and documentation until discharge, which all diagnostically eligible patients had. 

Data collection

Investigators extracted relevant patient data from paper medical records, including demographics (i.e. age, sex, weight, tobacco use), medical history (i.e. pre-existing conditions, allergies, routine medication), vital signs (i.e. respiratory rate, pulse rate, blood pressure, body temperature), initial and evolving clinical presentation from systematic clinical examination (e.g. respiratory, ophthalmological, intestinal, dermatological, neurological, psychological signs and symptoms), imaging results (i.e. chest x-ray, electrocardiogram), clinical management (i.e. oxygen, bronchodilation, hydrocortisone, antiemetics, dexamethasone, intubation), and outcomes (i.e. hospital admission, ICU admission, length of stay, recovery, death).

Analysis

We analyzed data using IBM Corp. Released 2015. IBM SPSS Statistics for Windows, Version 23.0. Armonk, NY: IBM Corp. and StataCorp. 2017. Stata Statistical Software: Release 15. College Station, TX: StataCorp LLC. We calculated descriptive statistics, as frequencies and proportions for discrete variables and measures of central tendency and dispersion (i.e. median, standard deviation, range) for continuous variables. As only one patient was admitted to ICU and died, we examined associations between demographic, clinical variables, hospital admissions, date, and other variables between the two attacks. As the sample was too small for multiple logistic regression, we calculated t-tests for continuous variables or Fisher’s exact test for categorical variables, using the p<0.05 significance threshold. 

Ethics 

The study obtained ethics approval from the Hama Health Directorate and the Ethics Committee of the Universitair Ziekenhuis Brussel (OG 016, approval number BUN143201837060). All participants or legal guardians provided oral informed consent before their records were included and all data were anonymized according to Helsinki Declaration principles. 

## Results

Participant demographics

Table 1 provides patient results. Fifteen patients presented at Kafr Zita Hospital on their day of exposure to toxic gas, seven on 11 April 2014 and eight on 22 May 2014. All seven presenting on 11 April were admitted, while only one of eight presenting on 22 May was admitted. Except for attack date, there were no significant associations between demographics and hospital admission or attack date. Similarly, except for one 35-year-old male patient with obesity, tobacco addiction, and arterial hypertension, who was hospitalized on 11 April for four days, there were no apparent associations between demographics and length of hospital stay.

**Table 1:** Chlorine-exposed patient results by hospital admission status (n=15) NB: *significant at 0.05 level.

Demographics		Not hospitalised, n=7 (%)	Hospitalised, n=8 (%)	Total, n=15 (%)
Attack	11-Apr-14	0 (0)	7 (88)	7 (47)
	22-May-14	7 (100)	1 (13)	8 (53)
Age (years)	Mean (range)	22.6 (SE 3.08)	28.5 (SE 7.02)	25.7 (2-59)
	under 15	1 (14)	2 (25)	3 (20)
	16-45	6 (86)	4 (50)	10 (67)
	46+	0 (0)	2 (25)	2 (13)
Sex	Female	3 (43)	4 (50)	7 (47)
	Male	4 (57)	4 (50)	8 (53)
Marital status	Unmarried	5 (71)	2 (25)	7 (47)
	Married	2 (29)	6 (75)	8 (53)
Weight (kg)	Mean (range)	66.7 (SE2.07)	64.8 (SE10.9)	65.7 (15-100)
Tobacco use	Non-smoker	6 (86)	5 (63)	11 (73)
	Smoker	1 (14)	3 (37)	4 (27)
Pre-existing disease	None	7 (100)	5 (63)	12 (80)
	Arterial hypertension	0 (0)	1 (13)	1 (7)
	Obesity	0 (0)	2 (25)	2 (13)
Vital parameters				
Respiratory rate	Mean (range)	44.9 (SE0.74)	43.3 (SE2.2)	44.0 (35-54)
	Tachypnoea	7 (100)	8 (100)	15 (100)
Pulse (BPM)*	Mean (range)	123.6 (SE1.9)	137.9 (SE5.7)	131.2 (115-160)
	Tachycardia	7 (100)	8 (100)	15 (100)
Blood pressure	Hypotension	7 (100)	6 (100)	13 (100)
(mmHg)	Not registered	0	2	2 (13)
	Mean (range)	82/56 (75/50-90/60)	67/47 (40/20-80/60)	74/52 (40/20-90/60)
Temperature (°C)*	Mean (range)	37.0 (SE0.14)	36.5 (SE0.09)	36.7 (36.0-37.5)
Signs and symptoms				
Respiratory	Dyspnoea	7 (100)	8 (100)	15 (100)
	Oral froth	7 (100)	8 (100)	15 (100)
	Feeling suffocated	7 (100)	7 (88)	14 (93)
	Rales	7 (100)	8 (100)	15 (100)
	Wheezing	7 (100)	8 (100)	15 (100)
	Intercostal retractions	7 (100)	8 (100)	15 (100)
	Central cyanosis	7 (100)	8 (100)	15 (100)
	Cough	7 (100)	8 (100)	15 (100)
Intestinal	Nausea	7 (100)	8 (100)	15 (100)
	Bloody vomiting	7 (100)	8 (100)	15 (100)
	Abdominal pain	7 (100)	8 (100)	15 (100)
Neurological	Muscle spasms	7 (100)	8 (100)	15 (100)
	Dizziness	7 (100)	6 (75)	13 (87)
	Fully conscious	7 (100)	8 (100)	15 (100)
	Normal pupils	7 (100)	8 (100)	15 (100)
Psychological	Irritation	7 (100)	8 (100)	15 (100)
	Anxiety	7 (100)	7 (88)	14 (93)
Dermatological	Pruritus	7 (100)	7 (88)	14 (93)
	Urticaria	7 (100)	8 (100)	15 (100)
Ophthalmological	Itchy eyes	7 (100)	7 (88)	14 (93)
	Excessive lacrimation	7 (100)	8 (100)	15 (100)
Exam				
Chest X-ray	nonspecific pulmonary infiltrates	7 (100)	8 (100)	15 (100)
Electrocardiogram	Sinus rhythm	7 (100)	8 (100)	15 (100)
	Sinus tachycardia	7 (100)	8 (100)	15 (100)
Treatment				
Oxygen		7 (100)	8 (100)	15 (100)
Hydrocortisone		7 (100)	7 (88)	14 (93)
Bronchodilators		7 (100)	8 (100)	15 (100)
Antiemetics		4 (57)	8 (100)	12 (80)
Dexamethasone		2 (29)	0 (0)	2 (13)
ICU/Intubation		0 (0)	1 (13)	1 (7)
Outcome				
Days hospitalised	Mean (range)	0 (0)	2.1 (SE0.67)	2.1 (1-6)
Death		0 (0)	1 (13)	1 (7)

Signs and symptoms

All patients suffered from tachypnoea, tachycardia, and hypotension, though temperatures were in the normal range. Of the three children, one 14-year-old had BP 80/60 mmHg while the other two (aged two and four years) could not be measured, indicating they were also hypotensive. All patients presented with the full spectrum of respiratory distress, including nonspecific pulmonary infiltrates on chest x-ray, sinus rhythm and tachycardia confirmed by ECG, intestinal distress, generalized muscle spasms, irritation, urticaria, and excess lacrimation. All remained fully conscious, with no registration of altered consciousness or changed pupil diameter. Most patients also suffered from other signs of chlorine intoxication such as mentioned in Table 1. 

As symptom intensity was generally not recorded, there were few associations between signs and symptoms, hospital admission, length of stay, or attack date. Mean pulse beats per minute (i.e. 138 versus 124; p=0.043) and body temperature (37.0 versus 36.5; p=0.019) were significantly higher among admitted patients than non-admitted. 

Treatment outcomes

All patients needed oxygen and bronchodilators, while 14 received hydrocortisone, 12 received antiemetics, and two received dexamethasone. Seven (47%) improved within hours of treatment and were discharged from the emergency department within 24 hours of admission. Eight (53%) required hospital admission, for a median stay of 2.1 days (range 1-6). Admission decisions depended on the clinically determined intensity of respiratory, gastrointestinal, and neurological symptoms. Twice as many hospitalized as non-hospitalized patients received antiemetics (eight versus four, while only non-hospitalized patients received dexamethasone (0 versus two). However, the numbers were too small to determine significance. 

There Was No Correlation Between the Use of Hydrocortisone and Length of Stay

All but one patient were discharged home in good general condition and with the apparent absence of complications. Only one, a 25-year-old female, required intubation and mechanical ventilation for which she was admitted to the ICU. She had no complaints of suffocation, nausea, abdominal pain, or dizziness recorded prior to ICU admission. She weighed 65 kilograms, blood pressure 60/40, pulse 130, and temperature 36.5°C. She received hydrocortisone and an antiemetic but no dexamethasone and was consecutively transferred to an ICU in Turkey, where she died four days later due to severe ARDS [22]. 

## Discussion

Implications of main results

This is the first analysis of the clinical presentation and management of weaponized chlorine-exposed patients in a conflict-affected hospital to the best of our knowledge. Govier et. al. has conducted a systematic review on civilian exposure to chlorine gas, none of the articles included documented weaponized chlorine gas attack [24]. Cevik et. al. reported military exposure of mass casualties from acute inhalation of chlorine gas as well [25].

Though the use of weaponized chlorine gas is prohibited internationally and constitutes a war crime, attacks appear to be happening in Syria and it is important for clinicians to know how best to respond, even in minimally resourced facilities. This study describes the presentation, treatment, hospital course, and outcome of patients exposed to weaponized chlorine gas in opposition-controlled Syria, indicating expected clinical presentation and trajectory in a large population with limited healthcare infrastructure. First, signs and symptoms in all victims were very similar during both attacks, indicating patients had been exposed to the same pathogenic factor. Second, most symptoms were of respiratory, digestive, and neurological origin, and the clinical state of most patients improved quickly, indicating patients had been exposed to chlorine gas. 

All exposed patients developed significant respiratory disorders, indicating severe airway inflammation, as well as digestive symptoms, suggesting gastrointestinal inflammation, and some degree of nervous system disorders. Management, which included symptomatic treatment using oxygen, inhaled bronchodilators, and intravenous corticosteroids, appeared effective given the poor medical infrastructure and resources available. Other inexpensive and simple medications, e.g. sodium bicarbonate, may enhance outcomes and could also be considered [26-28].

In most patients resolution of signs and symptoms was swift, indicating that even critically ill patients can often be discharged within a relatively short period after supportive care. However, one of 15 patients in this sample required mechanical ventilation. ICU beds are often limited in low-income and conflict-affected areas, challenging critically ill patient management and demonstrating the need for humanitarian partners to provide training and a well-equipped ambulance system for patient referral. People's first reaction to bombing is using basements as shelters which typically offer some protection from explosive attacks like barrel bombs, however, they are often unaware that in the case of chemical attacks, where toxic gases are heavier than air, they become at an increased risk of death. Thus it becomes of high priority to provide awareness campaigns to the public and training to the health providers. Barriers to performing effective campaigns include disrupted infrastructure, population displacement, lack of educational resources, and limited communication and movement [3].

Although most people exposed to chlorine gas will recover within months, and experience little or no residual dysfunction, the literature indicates that exposure may cause permanently impaired lung function, or lead to the development of persistent, nonspecific bronchial hyperreactivity [14, 29-31]. This implies that it is crucial for the international community to work harder against the use of chlorine gas and other chemical weapons during conflict and that exposed patients should be periodically reevaluated to ensure any long-term issues can be managed.

The international community's moral condemnation of chemical attacks tends to be useless if not supported by active prevention and lawful consequences to the perpetrators. Active prevention means preventing the production of the materials by reducing access to resources, however, this is hard to implement in the case of chlorine gas which is not very expensive to produce. 

The United Nations Security Council's (UNSC) investigations should continue and put lawful consequences in place to hold the Syrian government accountable for their actions in order not to make the use of chemical weapons normative in other conflict settings [3].

Limitations 

Several study limitations should be noted, most due to complex working conditions. First, our sample size is very small and only includes approximately 20% of those exposed, as it was neither practical nor ethically appropriate to include everyone. However, given the lack of data on weaponized chlorine exposure during the conflict, we considered it important to disseminate despite research limitations. Second, health workers obviously prioritized treating patients over clinical documentation, which limited available data. Third, it is difficult to document all casualties during conflict, which may have introduced some survivor bias, but Kafr Zita Hospital was the closest medical facility to both incidents and publicly available documentation suggests our findings are similar. Finally, interrater variability in recognizing and interpreting clinical signs, imaging results, or other variables, may have led to some misclassification though this was unlikely to have changed results noticeably. 

## Conclusions

Our results demonstrated that even in low-resource humanitarian settings the survival rate for chlorine gas exposed patients is fair, particularly if basic symptomatic treatment is performed with oxygen, bronchodilators, and corticosteroids. Despite the small sample, this study provides insight into clinical presentation, management, and outcomes of weaponised chlorine gas exposure. Further research is required to understand the chronic consequences of such toxic exposure.

## Appendices

SUPPLEMENTARY MATERIAL

Appendix 1

**Table 2:** Composition of medical point for dealing with chemical incidents in Kafr Zita’s Hospital

Decontamination and water supply unit	Medicines	Airway Management equipment	Intravenous kits
Water tank 10000 litres	atropine (ampulla)	Suction tubes, different sizes	IV Cannula, different sizes
Water Container 125 litres with tap	pralidoxime (ampulla)	Suction device	Syringe, different sizes
Bucket 30 litres	hydrocortisone (ampulla)	Oxygen generator	Plaster (25)
Jerry can 25 litres for solution preparation	ondansetron (ampulla)	Oxygen cylinder 40 litres	Sodium chloride solution (500 ml)
Water pipe with 4 shower points	diazepam (ampulla)	Oxygen mask with nebulizer	Sodium chloride solution (500 ml)
Bag transparent lockable 5 litres	salbutamol nebulizer solution	Pulse oximeter	IV Device
Bag refuse 100 litres black 70 microns	budesonide nebulizer solution	Bag valve mask adult	
TIE self-locking head plastic black 6 x 300 mm		Bag valve mask child	
Container for garbage with cover and wheels		Laryngoscope	
Plastic box transparent with cover		Endotracheal tube, different sizes	
Brooms		Video assisted Intubation device	
Shovel with handle			
Plastic sheeting 60x4 meter			
Electrical connections with 3 lamps			
Safety lamp torch rechargeable			
Foldable stretcher aluminium with 4 feet 220x58 cm			
Scrubbing brush soft bristles with handle			
Sponge			
Soap liquid 10 litres			
Cutter retractable with snap-off blade 18 mm			
Talc powder 20 kg			
Scissors 20 cm			
Adhesive tape roll			
Battery powered megaphone 6 Watt or more			
Toxic warning signal in plastic			
Decontamination check list in Arabic and plasticised			
Paint spray red fluorescent			
Personal protection equipment level C			
Replacement clothes for after decontamination			
Decontamination check list in Arabic and plasticised			
Paint spray red fluorescent			
Personal protection equipment level C			
Replacement clothes for after decontamination			

Appendix 2  

**Table 3:** Details on Kafr Zita’s Hospital Staff

General Surgery Physician	1
Internal medicine and Anaesthetist	1
General Practitioner	1
Pharmacist	1
Pharmacy technician	1
Laboratory technician	2
Radiology technician	2
Nurses	4 (2 men, 2 women)
Midwife	1
Intensive Care Technician	1
Operating Technician	2
